# New Passive Instruments Developed for Ocean Monitoring at the Remote Sensing Lab—Universitat Politècnica de Catalunya

**DOI:** 10.3390/s91210171

**Published:** 2009-12-14

**Authors:** Adriano Camps, Xavier Bosch-Lluis, Isaac Ramos-Perez, Juan F. Marchán-Hernández, Nereida Rodríguez, Enric Valencia, Jose M. Tarongi, Albert Aguasca, René Acevo

**Affiliations:** Department of Signal Theory and Communications, Universitat Politècnica de Catalunya & IEEC/CRAE-UPC Campus Nord D3, E-08034 Barcelona, Spain; E-Mails: xavier.bosch@tsc.upc.edu (X.B.-L.); isaacramos@tsc.upc.edu (I.R.-P.); jfmarchan@tsc.upc.edu (J.F.M.-H.); nereida@tsc.upc.edu (N.R.); valencia@tsc.upc.edu (E.V.); jose_miguel_tarongi@tsc.upc.edu (J.M.T.); aguasca@tsc.upc.edu (A.A.); rene.acevo@tsc.upc.edu (R.A.)

**Keywords:** microwave radiometers, global navigation satellite reflectometers, GNSS-R, ocean, salinity, sea state, payload

## Abstract

Lack of frequent and global observations from space is currently a limiting factor in many Earth Observation (EO) missions. Two potential techniques that have been proposed nowadays are: (1) the use of satellite constellations, and (2) the use of Global Navigation Satellite Signals (GNSS) as signals of opportunity (no transmitter required). Reflectometry using GNSS opportunity signals (GNSS-R) was originally proposed in 1993 by Martin-Neira (ESA-ESTEC) for altimetry applications, but later its use for wind speed determination has been proposed, and more recently to perform the sea state correction required in sea surface salinity retrievals by means of L-band microwave radiometry (T_B_). At present, two EO space-borne missions are currently planned to be launched in the near future: (1) ESA's SMOS mission, using a Y-shaped synthetic aperture radiometer, launch date November 2nd, 2009, and (2) NASA-CONAE AQUARIUS/SAC-D mission, using a three beam push-broom radiometer. In the SMOS mission, the multi-angle observation capabilities allow to simultaneously retrieve not only the surface salinity, but also the surface temperature and an “effective” wind speed that minimizes the differences between observations and models. In AQUARIUS, an L-band scatterometer measuring the radar backscatter (σ_0_) will be used to perform the necessary sea state corrections. However, none of these approaches are fully satisfactory, since the effective wind speed captures some sea surface roughness effects, at the expense of introducing another variable to be retrieved, and on the other hand the plots (T_B_-σ_0_) present a large scattering. In 2003, the Passive Advance Unit for ocean monitoring (*PAU*) project was proposed to the European Science Foundation in the frame of the EUropean Young Investigator Awards (EURYI) to test the feasibility of GNSS-R over the sea surface to make sea state measurements and perform the correction of the L-band brightness temperature. This paper: (1) provides an overview of the Physics of the L-band radiometric and GNSS reflectometric observations over the ocean, (2) describes the instrumentation that has been (is being) developed in the frame of the EURYI-funded *PAU* project, (3) the ground-based measurements carried out so far, and their interpretation in view of placing a GNSS-reflectometer as secondary payload in future SMOS follow-on missions.

## 1. Introduction: Principles of L-Band Microwave Radiometry and GNSS-R over the Ocean

Sea Surface Salinity (SSS) is a key climatologic and oceanographic parameter. It has a significant influence on ocean currents (thermo-haline circulation), it is related to the evaporation minus precipitation, is responsible for El Niño Southern Oscillation (ENSO) events, and determines the life conditions of some species…

Sea surface salinity can be determined by means of microwave radiometry. The largest sensitivity is found at P-band [1], but at L-band (1.400–1.427 MHz) there is a quiet frequency band where the sensitivity to SSS is still relatively “high” (∼0.5 K/psu at 15 °C). However, the sea emission in the L-band depends on other parameters, such as the Sea Surface Temperature (SST) and mainly, the sea state (surface roughness) [2]. Furthermore, the relationship between the brightness temperature changes and the sea state is very complex, since the sea emission is not dominated either by capillary waves, nor by the long waves, and even swell effects have an influence.

**Figure 1.**
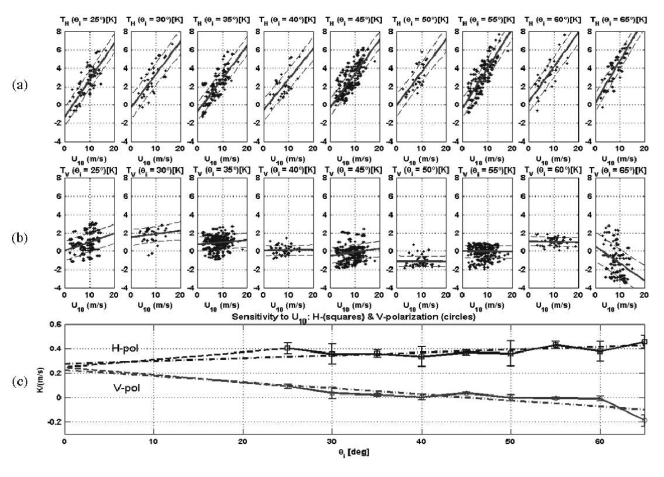
Derivation of the brightness temperature sensitivity to wind speed. Scatter plots of the brightness temperature change at (a) horizontal (ΔT_B h_) and (b) vertical (ΔT_B v_) polarizations *vs.* the 10-m height wind speed (U_10_). Solid line: linear fit, and dashed lines: percentile 50% as a function of U_10_ for incidence angles from 25° to 65°. (c) Derived T_B h,v_ sensitivity to wind speed as a function of (solid line) polarization and incidence angle, associated ±1 σ error bars, and (dashed lines) linear fit. All data points used (Figure 6 from [3]).

Experimental activities conducted in the frame of the SMOS mission have shown that regressions in terms of the geophysical variables usually measured such as the wind speed (WS) and/or the significant wave height (SWH), exhibit too high a scattering (Figure 1) to provide a satisfactory correction to perform the sea state correction for the salinity retrieval, and that it is advisable to retrieve an “effective” wind speed as well, that can compensate for modeling errors [3].

Within the frame of the EURYI program, in 2003 the *PAU* (*Passive Advanced Unit for ocean monitoring*) project was proposed to the European Science Foundation to test the feasibility of using GNSS-R over the sea surface to make sea state measurements and, jointly with IR observations to obtain the SST, perform the corrections of the L-band brightness temperature [4]. GNSS-R was originally devised for altimetry applications [5], and here it is extended to try to obtain a direct correction for the sea state, without having to rely neither in numerical sea surface spectra models, nor in scattering and emission models.

**Figure 2.**
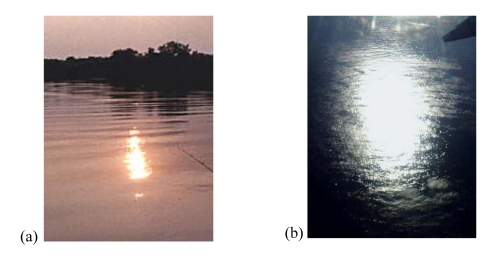
Sun glint over the sea under: (a) calm and (b) windy conditions.

The concept is simple: when the electromagnetic wave is scattered over the sea surface, the scattered signal changes its polarization (from RHCP to mostly LHCP) and arrives at the receiver mainly from the specular reflection point, determined by the shortest distance between the transmitting GPS satellite and the receiver, but when the sea is roughed, the scattered signals come from a wider region (known as “glistening zone”) that enlarges with increasing sea state, in a similar manner as the Sun reflecting over the sea (Figure 2).

**Figure 3.**
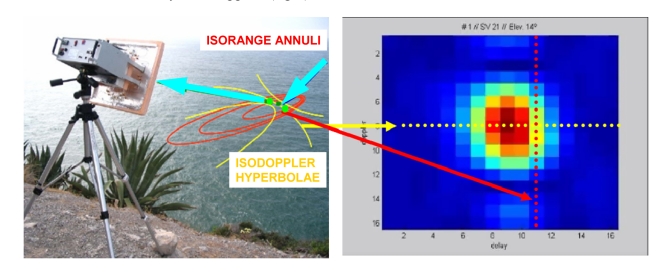
(left) GNSS-R geometry of observation from Garraf coast (South of Barcelona). Red: isorange annuli (same delay), Yellow: isoDoppler hyperbolae (same Doppler), Green dots with same delay and Doppler. (right) measured DDM.

When observing the GNSS reflected signals, two points over the sea surface correspond to the same delay and Doppler coordinates, with minimum delay corresponding to the specular reflection point (Figure 3). In *PAU* it was proposed to measure the complete Doppler-Delay Maps (DDMs) to make the sea state correction of the brightness temperature required for the salinity retrieval. The DDM is the square of the absolute value of the correlations of the reflected GNSS signals with local replicas of the transmitted signal, but shifted in delay and Doppler, and it is given by [6]:

(1) $${\left|\text{DDM}(\Delta \tau ,\Delta f_{d})\right|}^{2}=T_{1}^{2}\iint \frac{{\left|R\right|}^{2}D^{2}(\vec{\rho })\Lambda^{2}(\Delta \tau ){\left|S(\Delta f_{d})\right|}^{2}}{4R_{0}^{2}(\vec{\rho })R^{2}(\vec{\rho })}\frac{q^{4}(\vec{\rho })}{q_{z}^{4}(\vec{\rho })}P_{v}\left(\frac{{\vec{q}}_{⊥}}{q_{z}}\right)d^{2}\vec{r}$$

where *T_i_* is in the integration time, *R* is the Fresnel reflection coefficient, *D* is the directivity of the receiving antenna, *χ*(Δ*τ,* Δ*f_d_*) ≈ Λ (Δ*τ*) · *S*(Δ*f_d_*) is the Woodward ambiguity function, that can be approximated by the product of a triangle function Λ (Δ*τ*) = (1 − |Δ*τ*||*T_c_*) if |Δ*τ*|/*T_c_*, being *T_c_* the chip period, and a sinc function *S*(Δ*f*) = exp(−*j π* Δ*f T_i_*) · (*π* Δ*f T_i_*)/(*π* Δ*f T_i_*), *R*_0_ (*ρ⃗*) and *R*_0_ (*ρ⃗*) are the distances between the transmitter and receiver to the scattering point, *q* is the amplitude of the scattering vector *q⃗* ≜ *k*(*n̂* − *m̂*) where *k* is the wavenumber, *n̂* and *m̂* are the unit vectors of the incident and scattered waves, respectively, *q_z_* is the *z* component of *q⃗*, and *q⃗_⊥_* is the perpendicular component, and *P_v_* is the sea surface slopes probability density function, and the integral is performed over the whole glistening zone.

Measuring the whole DDM, avoids the loss of information due to the asymmetry of the DDM originated by the relative orientation of the transmitter, the receiver and the wind direction, that is not captured when only the so-called *waveforms* (time domain correlation when the Doppler shift is compensated) are measured (*waveform* = DDM(Δ*τ,* Δ*f_d_* = 0)).

**Figure 4.**
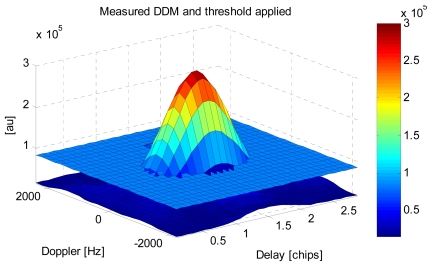
Measured (not normalized) 1 s incoherently integrated DDM with the threshold applied to compute its volume. Noise is well below the threshold [9].

The volume of the normalized DDM (peak amplitude equal to 1) above a given threshold (Figure 4) has been found to provide a measurement of the area over which the GNSS signals are scattered, and it can be related to geophysical variables such as the sea surface roughness, without need of any intermediate model, either numerical to compute the scattering or for the sea surface spectrum [7,8].

## 2. Instrument Developments

In order to test the feasibility of using GNSS-R over the sea surface to make sea state corrections to the radiometric data, the *PAU* (*Passive Advanced Unit for ocean monitoring*) project was proposed to the European Science Foundation in 2003, and it was granted in 2004 within the EURYI program. *PAU* is also a test bed of new technological demonstrators such as real aperture radiometers with digital beamforming and polarization synthesis, and fully-digital synthetic aperture radiometers *etc.* In the *PAU* system a suite of three different instruments operate in a synergetic way:

1. *PAU/RAD*: an L-band radiometer to measure the brightness temperature of the sea surface,
2. *PAU/GNSS-R*: a GPS-reflectometer using the L1 C/A code to measure the sea state, and
3. *PAU/IR*: two infrared radiometers to measure the sea surface temperature,

and both *PAU/RAD* and *PAU/GNSS-R* share the same RF front-end.

A number of *PAU* system prototypes have been developed:

1. *PAU-Real Aperture* instrument with a 4 × 4 element array with digital beamforming and polarization synthesis that uses of an innovative pseudo-correlation radiometer topology to avoid the classical input switch in a Dicke radiometer and
2. *PAU-Synthetic Aperture* instrument, which is also used to test potential new technologically developments and algorithms for future SMOS missions.
  In addition to these two, and in order to advance the scientific studies relating the GNSS-R and radiometric observables other *PAU* demonstrators have been developed:
3. *PAU-OR* with just one element for ground tests and algorithms development, and *griPAU*, an improved *PAU-OR* instruments fully automated,
4. *PAU-ORA,* a lighter version of *PAU-OR* for aircraft operations from a remote controlled plane, and
5. *MERITXELL* (*Multi-frequency Experimental Radiometer With Interference Tracking For Experiments Over Land And Littoral*) a classical Dicke radiometer, that includes not only L-band, but S-, C- X-, K-, Ka-, and W-bands, plus a multi-spectral camera, in addition to the *PAU/IR* and *PAU/GNSS-R* units.

In these technology demonstrators the input signals (*PAU/RAD* and *PAU/GNSS-R*) are the same: the L1-GPS band. This is not critical, since, due to the scattering on the sea surface, the scattered GPS signal is at least 23 dB below of the thermal noise signal (to be measured by *PAU/RAD*). Thanks to the 30.1 dB correlation gain, *PAU/GNSS-R* can detect the GPS signal when the correct C/A code is applied, and the error introduced in *PAU-RAD* observables is negligible, and it only occurs when the specular reflection is coming from the antenna boresight. Taking into account that the beam can be electronically steered the chance of having a significant interference is very remote.

Signals' bandwidth are limited to the GPS bandwidth (2.2 MHz) and are demodulated at an intermediate frequency (IF) of 4.309 MHz. Signals are then digitalized at 8 bits at a sampling frequency of 5.745 MHz, to allow the use of digital demodulation, using band-pass sampling techniques.

These different instruments are explained in the following sections.

### 2.1. PAU-Real Aperture

**Figure 5.**
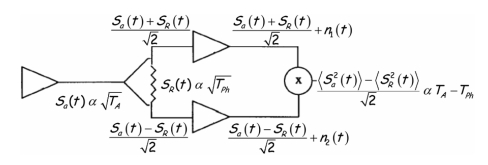
Block diagram of the *PAU/RAD* concept with just one receiver.

One of the technological goals of the project is to demonstrate the feasibility of combining in a single hardware two types of receivers: the radiometer itself (*PAU/RAD*) which, for stability reasons cannot be a total power radiometer, and the GNSS-Reflectometer (*PAU/GNSSR*). In order to be able to use the same receivers for both the radiometer and the GPS-reflectometer, a new radiometer topology has been devised (Figure 5). Another technological goal, as challenging as the first one, is to provide an L-band radiometer with a digital beamforming. This capability is a very useful novelty in microwave radiometry, since it allows one to obtain simultaneously brightness temperature measurements at different incidence angles without mechanical scan.

As compared to a real aperture radiometer, instead of connecting the antenna output directly to the radiometer receiver, it is connected to the input of a Wilkinson power splitter that divides the signal in two signals that are in phase. However, the 100 ohm resistor of the Wilkinson power splitter that connects the two outputs also adds two noise signals that are 180° out-of-phase.

Therefore, the signals at the input of the two channels of the radiometer are the sum and the difference of the antenna signal and the noise generated by the Wilkinson power splitter resistor, which is proportional to the physical temperature of the resistor. Once properly amplified, down-converted, and sampled they are finally cross-correlated leading to an output that is proportional to the difference between the antenna temperature and the physical temperature of the Wilkinson power splitter resistor. That is, the system output is the same as the one of the Dicke radiometer, but the input signal is not chopped, so that it can be used to track the GPS-reflected signal.

**Figure 6.**
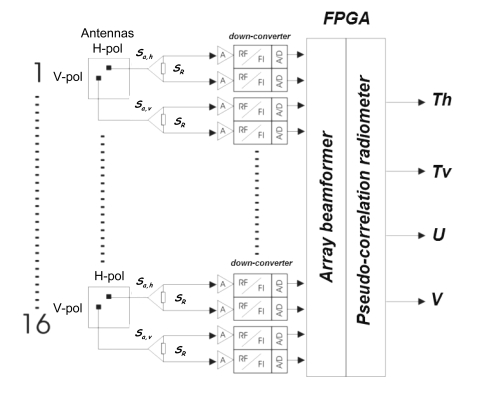
Schematic of the *PAU-Real Aperture* with 16 elements [4].

The extension to the *N*-element case is straightforward although technology challenging. Instead of one antenna, there are *N*-dual polarization antennas, whose outputs are connected each to a Wilkinson power splitter. After amplification, down-conversion, and sampling, the *N*-outputs at each polarization are combined with the ones from other receivers with the appropriate amplitude and phases so as to digitally form the beam(s). The final output of each beam is obtained by cross-correlating the upper and lower branches, corresponding to the sum and difference signals (Figure 5). If the vertical-polarization signals are cross-correlated, the antenna temperature at vertical polarization is measured. If the horizontal-polarization signals are cross-correlated, the antenna temperature at horizontal polarization is measured. And if the vertical-polarization signal is correlated with the horizontal-polarization one, the third and the fourth Stokes elements are measured, as twice the real and imaginary parts of the complex cross-correlation. Note that, since the vertical and horizontal channels do not share a Wilkinson power splitter, there is no “Dicke effect” (output proportional to the difference between the antenna temperature and the physical temperature of a reference) for the third and fourth Stokes parameters. Figure 6 shows the schematic of the whole *PAU-Real Aperture* instrument.

In addition, two calibration signals are required for calibration purposes:

- uncorrelated noise, generated by a matched load at each input channel, to compensate for instrumental biases (cross-correlations must be zero), and
- two different levels of correlated noise generated by a common noise source, to compensate for phase and amplitude mismatches among receivers.

**Figure 7.**
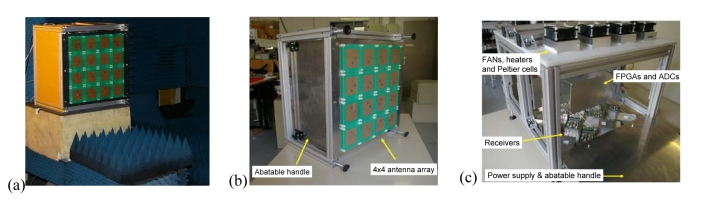
(a) *PAU-Real Aperture* without radome in UPC anechoic chamber for testing beamforming performance, (b) front view of the 4 × 4 element array, and (c) inside of the instrument showing the distribution of the dual-polarization receivers, FPGAs for data processing, and the temperature control.

Details on the calibration techniques and beamforming can be found in [10-12]. Figure 7 shows the *PAU-RA* instrument: (a) in the UPC anechoic chamber for testing, (b) front-view without cover, and (c) inside view without cover and with foldable sides opened.

### 2.2. PAU-Synthetic Aperture

The receivers and frequency plan of *PAU-Synthetic Aperture* is the same as for *PAU-Real Aperture*, but since the number of receiving elements (Y-shaped array with 8 elements per arm plus a central one) is much larger than in the *PAU-Real Aperture* case (16 elements), limitations in the FPGAs processing capabilities, forced to retain only one receiver per element and polarization, and the Wilkinson input switch was removed (topology in Figure 5 could not be followed).

Another of the objectives of PAU-Synthetic Aperture was to test new techniques, technologies and algorithms that could be eventually used in future SMOS missions. To better understand this, Table 1 summarizes the main parameters of the MIRAS instrument aboard SMOS mission [13] and the *PAU-Synthetic Aperture* ones. The rightmost column lists the rationale behind each technological decision.

**Figure 8.**
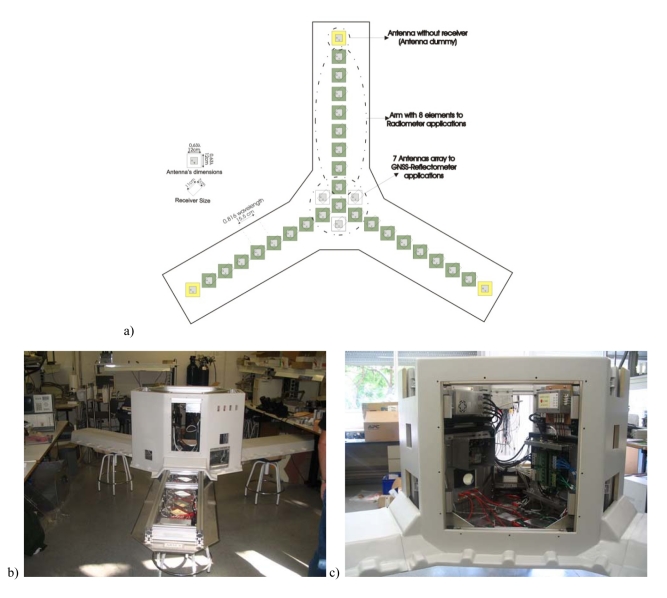
(a) *PAU*-Synthetic Aperture's topology, (b) View of the whole instrument with one arm opened, and (c) Detail of the hub, including digital correlator unit (right hand side, bottom), base-band clock distribution (right-hand side, top), calibration unit, including 2-level noise source and Pseudo-Random Noise (PRN) generator (left-hand side, bottom), power supply (left-hand side, up).

The *PAU*-Synthetic Aperture is composed by a Y-shaped array of eight antennas per arm plus the one in the center, and additional dummy antenna at the end of each arm. Moreover, the four central antennas plus three additional ones (seven in total) are used to create a steerable array for *PAU/GNSS-R* to point to the specular reflection points (total = 31 dual-polarization antennas). Figure 8 shows the *PAU*-Synthetic Aperture topology, and two figures taken during the integration. Hardware and simulation details can be found in [16,17].

### 2.3. PAU—One Receiver and griPAU

In order to develop the science behind the GNSS-R observables and their relationship with the brightness temperatures, two simplified instruments have been developed, which consist of just one LHCP down-looking antenna, a seven LHCP path hexagonal array, and a RHCP up-looking antenna.

Two of these instruments have been built, one for ground based operations (PAU-One Receiver–rev 1–[18] and griPAU–rev 2–[19]), and another one for airborne operations (PAU-One Receiver Airborne, see Section 2.4).

**Figure 9.**
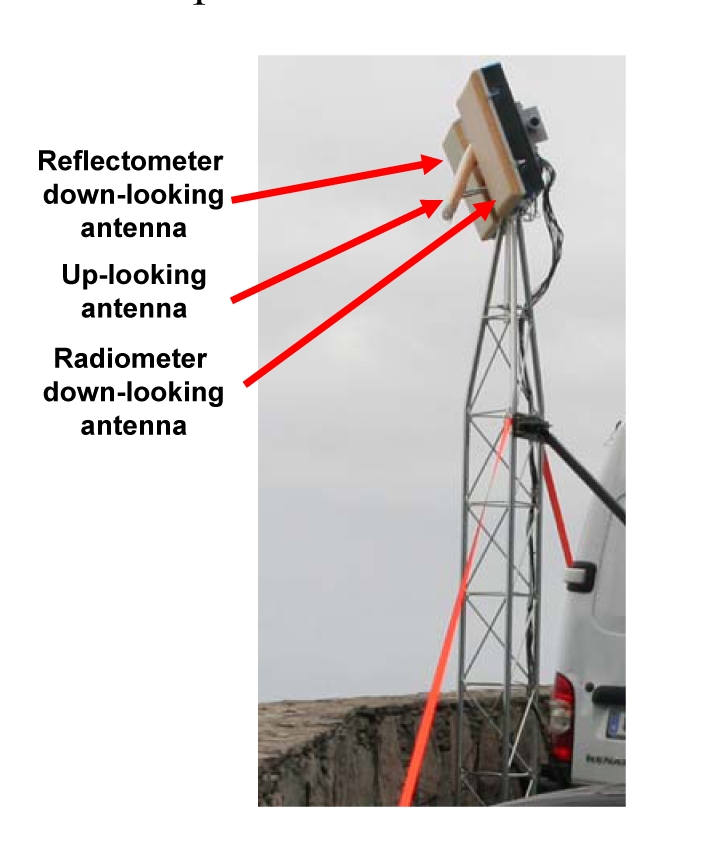
*griPAU* instrument mounted on a 6 m tower with automatic antenna positioner, during the ALBATROSS 2009 field experiment in Gran Canaria (Canary Islands, Spain).

**Figure 10.**
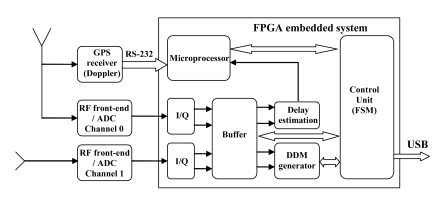
*griPAU* instrument block diagram showing: commercial GPS receiver to provide Doppler estimates, up-looking receiving element to provide delay estimates (every 5 ms), and down-looking receiving element (with 7 LHCP hexagonal patch array) to collect the reflected signal. The whole system is embedded in a Xilinx Virtex-4 FPGA and has serial USB connectivity.

Figure 9 shows the griPAU instrument deployed during the Advanced L-BAnd emissiviTy and Reflectivity Observations of the Sea Surface (ALBATROSS) 2009 field experiment in the Canary Islands. In this particular implementation of the PAU concept, two 7-patch hexagonal arrays are used: one for a dual polarization radiometer (vertical and horizontal polarizations) at 1.400–1.427 MHz (instead of f_0_ = 1575 MHz, B = 2.2 MHz), and the second one for PAU/GNSS-R. The smaller up-looking patch antenna in the center is used to track the delay of the direct signal, and fed it to the reflectometer. Figure 10 shows the griPAU (PAU/GNSS-R) block diagram. This instrument includes an automatic tracking of the specular reflection point of the pre-selected GPS satellite to simplify instrument's operation, while at the same time ensures observations collocated in time and space, exactly in the 1.400–1.427 MHz band used for passive observations.

### 2.4. PAU-One Receiver Airborne

**Figure 11.**
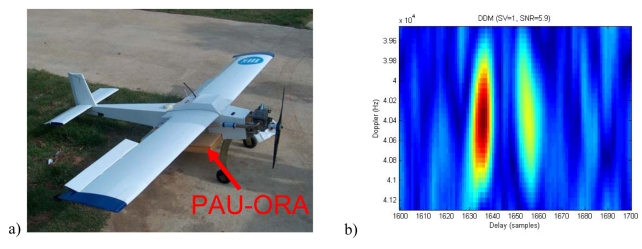

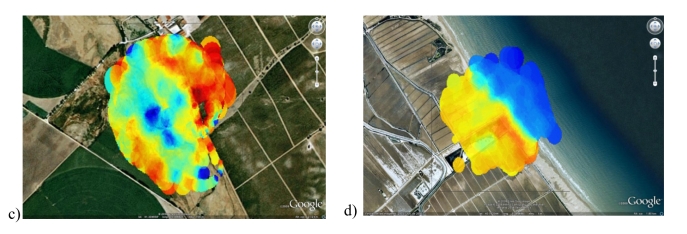
(a) *PAU*–One Receiver Airborne instrument block mounted on the bay of a Remote Control aircraft. (b) DDMs obtained collecting simultaneously the direct (RHCP) and reflected (LHCP) signals. Brightness temperature maps measured over (c) Vadillo de la Guareña (Zamora, Spain) and (d) Marquesa Beach (Ebro river mounth, Tarragona, Spain).

Figure 11a shows the *PAU*-One Receiver Airborne on the bay of a remote controlled aircraft. Details on the control, telemetry, data links and data storage can be found in [20-22]. Figure 11b shows the DDMs measured when the direct (RHCP) and reflected (LHCP) signals are collected simultaneously using two different antennas connected to the inputs of a non-resistive 2-way power combiner. The left-hand side peak corresponds to the direct signal, which has a larger amplitude, while the right-hand side one corresponds to the reflected one, which is attenuated in the scattering and in the longer signal path. The separation between peaks is 21 samples, which correspond to ∼770 m, since in this implementation of the instrument, the sampling frequency is 8.1838 MHz. Therefore, since the antenna was pointing to the nadir direction and the GPS satellite was close to the zenith, the estimated height is ∼385 m, which is very close to the flight height (379 m). This design offers several advantages over the previous ones (just measuring the reflected signal) since it intrinsically provides absolute calibration of the scattering coefficient (ratio of peaks between direct and reflected DDM), if offer altimetry capabilities, and sea state determination using the full DDM, as shown in Section 3.

### 2.5. The Multifrequency Experimental Radiometer with Interference Tracking for Experiments over Land and Littoral (MERITXELL)

The *MERITXELL* radiometer is a step forward advancing our understanding of the potentials of combining data from different sensors: microwave radiometers, multi-spectral and TIR cameras, and GNSS Reflectometers. It will also be used in testing radio frequency interference (RFI) detection and mitigation algorithms for microwave radiometry.

The *MERITXELL* microwave radiometer is a multi-band dual-polarization Dicke radiometer covering eight protected bands used for passive remote sensing: L, S, C, X, K, Ka, and W (see Table 2). To add flexibility and simplify the design, a spectrum analyzer is used as IF stage, for filtering and power detection for all bands. This allows an easy reconfiguration of the band and/or frequency response shape, since the antennas and amplifiers response exceed those indicated in table 2. Antennas are 4 × 4 dual-polarization path arrays at L, S, and C bands, and horn antennas with a lens in the aperture to provide a quasi Gaussian beam for the other bands.

In addition, *MERITXELL* includes a thermographic camera (320 × 240 pixels) operating in the 8–14 μm range, a multi-spectral camera (640 × 480 pixels) with four spectral bands: red (λ_0_ = 0.62 μm), green (λ_0_ = 0.54 μm), blue (λ_0_ = 0.45 μm) and Near Infra-Red (λ_0_ = 0.80 μm), and a *PAU/GNSS-R* unit.

**Figure 12.**
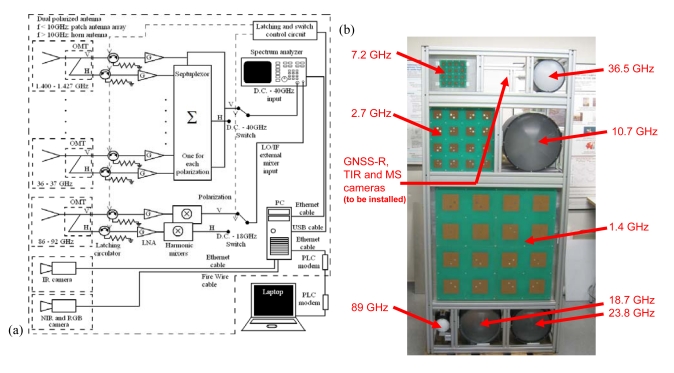
(a) *MERITXELL* microwave radiometer schematic, and (b) front view.

Figure 12a shows the schematic of the *MERITXELL* microwave radiometer, and Figure 12b shows the front view with the antennas mounted (cameras and *PAU/GNSS-R* will be placed in the hole in the center of the top row).

**Figure 13.**
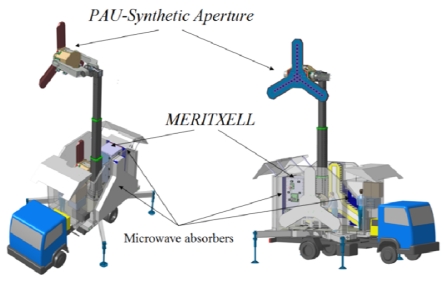
Artist's view of *PAU-Synthetic Aperture* and *MERITXELL* instruments on truck with 8 m robotic mast.

At the time of writing this manuscript the construction of a truck with a telescopic robotic mast to hold and orient both the *PAU*-Synthetic Aperture and MERITXELL instruments is being finished. It has been designed to hold one of these two instruments up to a 8 m height above the ground level, while the other is in the parking position (e.g., MERITXELL in Figure 13), and withstand up to 100 km/h wind loads. Figure 13 shows a view of the *PAU-Synthetic Aperture* instrument deployed, and the *MERITXELL* instrument in the parking position. Each has their own panel of microwave absorbers for calibration purposes.

## 3. Field Experiments

**Figure 14.**
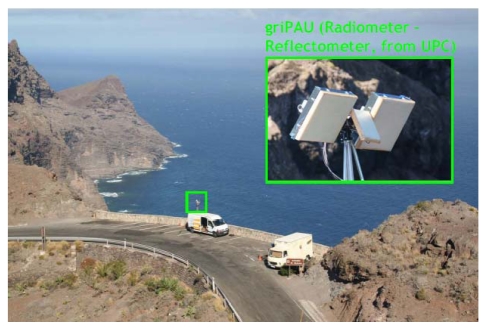
*griPAU* deployed during the ALBATROSS 2009 field experiment.

During May-June 2008 the first *PAU*-One Receiver was deployed at El Mirador del Balcón, La Aldea de San Nicolás, in the North-West coast of Gran Canaria in the Canary Islands, and gathered for the first time ever L-band radiometric and GNSS-R data, together with oceanographic data (sea surface temperature + sea surface directional spectrum buoys). The field experiment was repeated during the same period of time in 2009 with an improved version of the instrument (*griPAU*) that collected radiometric and GNSS reflectometric data collocated both in time and space using two different antennas with the same 22° beamwidth (Figure 14).

**Figure 15.**
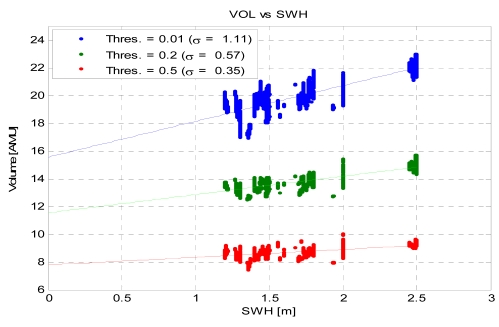
DDM-volume dependence on the SWH for three different thresholds [8].

Now, we start to understand the relationship between the sea state and the GNSS-R observables (DDMs) and the changes in the brightness temperature. Figure 15 shows the scatter plot of the measured DDM volume (in arbitrary units) *vs.* the SWH for different threshold values. It can be appreciated that increasing the threshold decreases the sensitivity to SWH since a lower volume is being considered. However, this threshold cannot be arbitrarily small, since it has to be above the noise threshold to provide meaningful observations (Figure 4).

**Figure 16.**
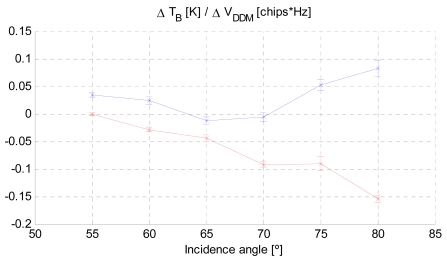
Estimated brightness temperature sensitivity to changes in the normalized DDM volume at vertical (red) and horizontal (blue) polarizations respecively [9].

The correlation of the instantaneous brightness temperature changes and the instantaneous DDM volumes observed during ALBATROSS 2009 is shown in Figure 16 for incidence angles larger than 55°, since the cliff already imposed a 45° mask, and incidence angles between 45° and 50° were affected by multi-path.

As it can be appreciated, the behavior is very similar to that shown in Figure 1, including a zero crossing around 55° degrees at vertical polarization, which suggests that both descriptors (WS in Figure 1 and the volume of the DDM, V_DDM_, in Fig 16) are measuring the same phenomena.

Despite these encouraging results, there is still a long way to go until meaningful physical quantities that can be successfully extracted from satellite data to be used by the oceanographic communities, and they can be used to perform the sea state correction in sea surface salinity retrievals. More extensive data sets need to be gathered and processed.

The limited GNSS-R data gathered by the UK-DMC satellite and publicly available [23] shows the potential of this technique, and supported the proposal of a *PAU* secondary payload in SeoSat/INGENIO (Spanish Earth Observation Satellite) [24]. This proposal went through phase A, but did not succeed to pass into phase B due to the accommodation issues with the primary payload raised after a configuration change. Simplified, lighter and less power consuming payloads are currently under development in cooperation with industry and they will be available for future launches of opportunity in micro- or even in pico-satellites.

## 4. Conclusions

This paper has presented the basic principles of GNSS reflectometry and how these observables can be eventually used to correct for the sea state induced changes in the brightness temperature at L-band. These corrections [2] are needed to improve the quality of the sea surface salinity retrievals using L-band microwave radiometry.

The suite of *PAU* instruments that has been (is being) developed at the Remote Sensing Lab of the Universitat Politècnica de Catalunya has been presented. These include: (1) *PAU-Real Aperture* with and array of 4 × 4 dual-polarization elements with digital beamforming and polarization synthesis, (2) *PAU-Synthetic Aperture* with a Y-shaped array of eight elements per arm to test digital technologies and new calibration techniques that can be eventually be used in future SMOS follow-on missions, (3) *PAU-One Receiver* and *griPAU*, one receiver only versions to demostrate the novel *PAU* radiometer topology concept (Figure 5) and perform round-based field experiments, (4) *PAU-One Receiver Airborne*, one receiver only version to demostrate the concept from remote controlled aircraft, and (5) *MERITXELL* a version with multi-frequency Dicke radiometers, multi-spectral and thermal infrared cameras.

These instruments have been developed for two purposes: (1) to analyze the nature of reflectometric observables and their relationship with the brightness temperature at L-band, and (2) to be technological demostrators of improvements to be applied in future space-borne missions (SMOS follow-on missions), or secondary payloads that can help in the sea state correction.

The promising results of two field experiments carried out in Gran Canaria, during 2008 and 2009 have been presented. However, these techniques are not restricted to the observation of the ocean, but they can also be applied over land to infer soil moisture and vegetation height [25,26].

## Figures and Tables

**Table 1.** MIRAS instrument and *PAU-Synthetic Aperture* main parameters.

**Parameter**	**MIRAS/SMOS**	**PAU-Synthetic Aperture**	**Rationale for changing the parameter**
**Frequency operation**	protected band: 1400–1427 MHz	GPS L1 (1575.42 MHz)	availability of integrated circuits
**Bandwidth**	19 MHz	2.2 MHz	limitation of integrated circuits and FPGAs minimizes spatial decorrelation.
**Arm size**	4 m	1.3 m	operation from a truck
**Altitude**	755 km	ground-based experiments	-
**Antenna type**	dual-polarization patch antenna (non simultaneous)	dual polarization patch antenna (simultaneous)	multiply by 2 the integration time eliminate polarization transitions
**Number of antennas per arm**	23	8+1 (dummy)	dummy antenna at edge for better pattern similarity
**Total number of antennas**	69	31	-
**Antenna spacing**	0.875 *λ* at 1400 MHz	0.816 *λ* at 1575.42 MHz	reduced antenna spacing to increase alias-free field-of-view double integration time
**Receiver type**	1 per element	2 per element (1 per polarization)	allow full-polarimetric mode without pol-switching schemes [14]
**Topology of the LO down-converter**	distributed LO (groups of 6 elements)	centralized reference clock + internal PLL in each receiver for LO generator.	avoid correlated noise from common LO leaking to outputs through mixers and generating correlation offset
**Quantization**	1 bit IF sampling	8 bit IF sub-sampling using a external ADC	use digital I/Q demodulation, low-pass filtering and power detection
**I/Q conversion**	analog	digital	avoid phase quadrature errors and different receivers' noise temperature between I/Q branches
**Frequency response shaped by…**	analog RF filter	digital low-pass filter	match global frequency response using narrow digital low-pass filters
**Power measurement system (PMS)**	analog, using detector diode	Digital (FPGA)	avoid detector diodes thermal drifts
**Digital Correlator Unit**	*f_CLK_* = *f_sample_*	*f_CLK_* ≫ *f_sample_*	use of band-pass sampling techniques
**Imaging capabilities**	dual-pol or full-pol (sequential)	Full-pol (non-sequential)	flexibility
**Calibration capabilities**	uncorrelated/2-level correlated noise injection	uncorrelated/2-level correlated noise injection and calibration using Pseudo-Random Noise (PRN) sequences	test new calibration algorithms based on the injection of Pseudo-Random Noise (PRN) sequences [15].
**Integration time**	1.2 s	1 s, 0.5 s, 100 ms and 10 ms	flexibility

**Table 2.** MERITXELL microwave radiometer bands and antenna parameters.

**Band**	**Central frequency**	**Bandwidth**	**Antenna beamwidth**	**Main beam efficiency**
L	1.4135 GHz	27 MHz	∼25°	98 %
S	2.695 GHz	10 MHz	∼25°	98 %
C	7.185 GHz	90 MHz	∼25°	98 %
X	10.69 GHz	20 MHz	∼5°	95 %
K	18.7 GHz	200 MHz	∼5°	95 %
K	23.8 GHz	400 MHz	∼5°	95 %
Ka	36.5 GHz	1 GHz	∼5°	95 %
W	89 GHz	6 GHz	∼5°	95 %
